# Supplementary figures and images for: Mitochondrial double-stranded RNA homeostasis depends on cell-cycle progression
Source: Life Sci Alliance. 2024 Aug 29;7(11):e202402764. doi: 10.26508/lsa.202402764 (PMC11361371; doi:10.26508/lsa.202402764)

Figure S1C

50µg total lysate / sample

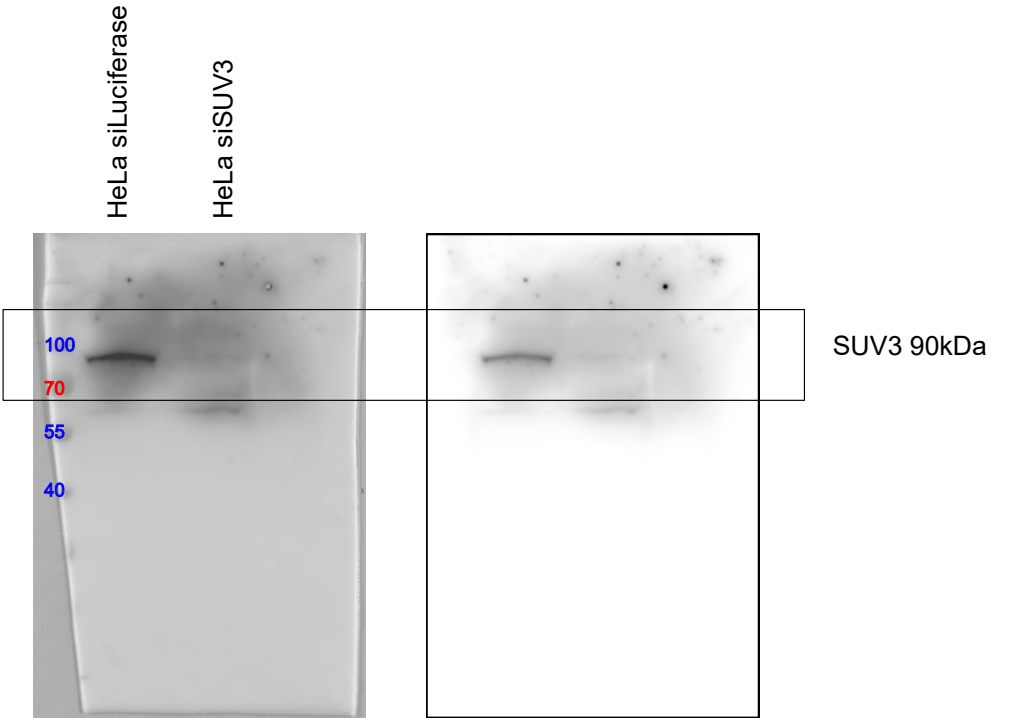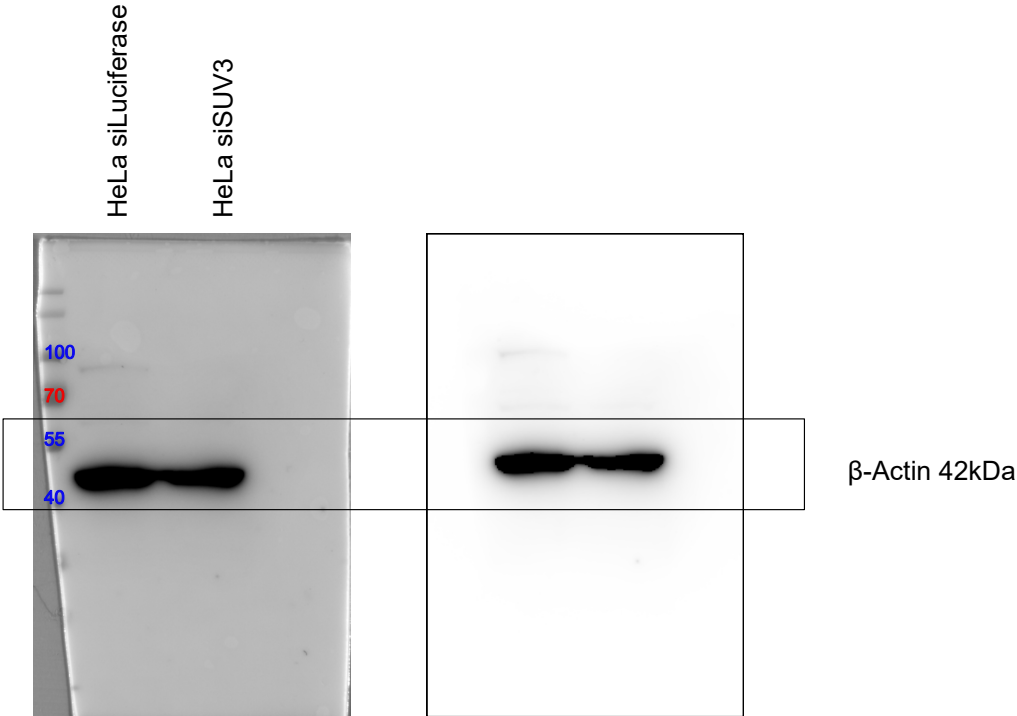

Supplement: Supplementary file 1 [file LSA-2024-02764_SdataFS1.pdf]

Figure S4B

Northern Blot

5µg total RNA / sample

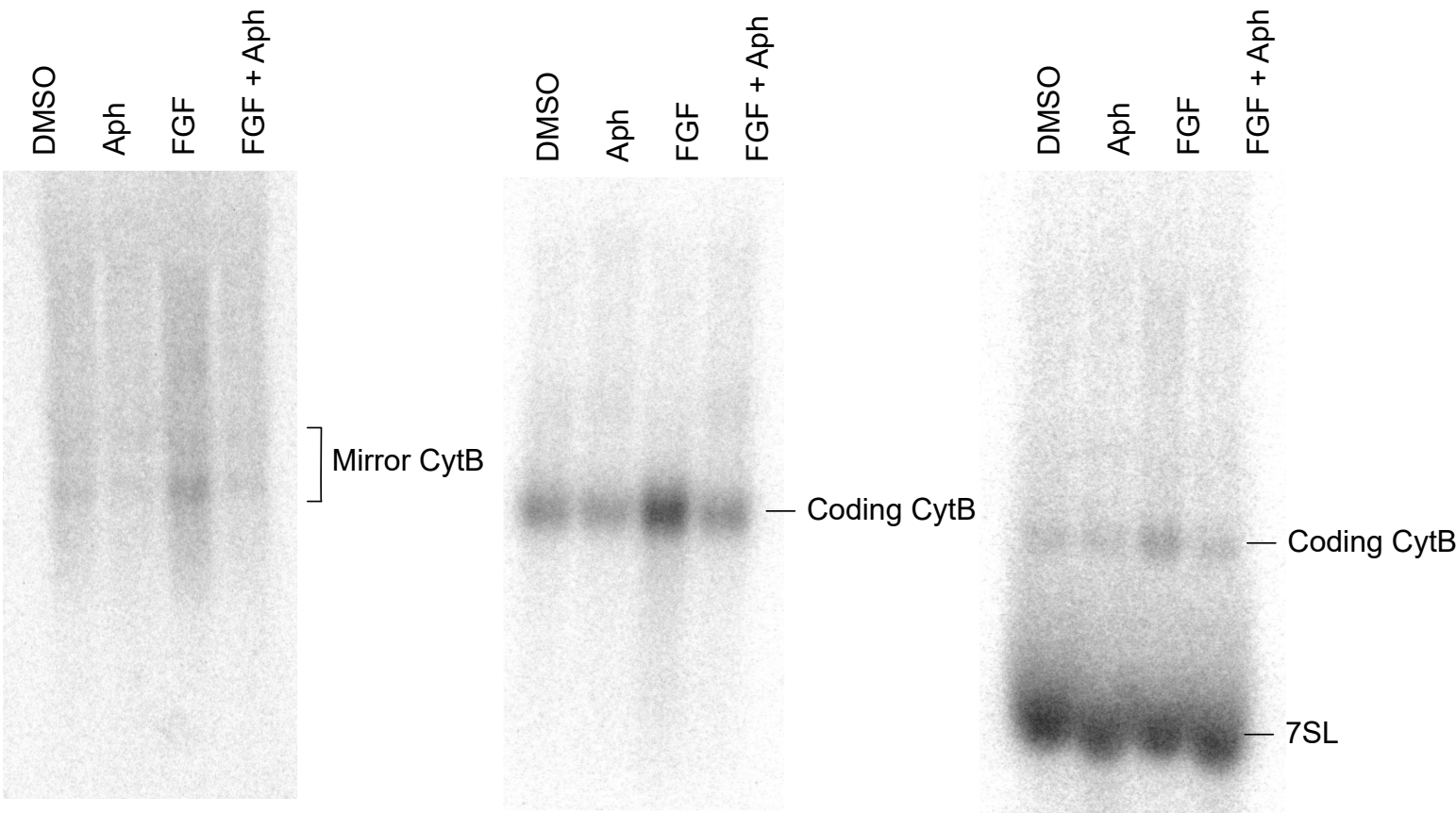

Supplement: Supplementary file 5 [file LSA-2024-02764_SdataFS4.pdf]
