# Supplementary material for: Mitochondrial double-stranded RNA homeostasis depends on cell-cycle progression
Source: Life Sci Alliance. 2024 Aug 29;7(11):e202402764. doi: 10.26508/lsa.202402764 (PMC11361371; doi:10.26508/lsa.202402764)
Supplement: Supplementary file 2 [file LSA-2024-02764_SdataFS2.pdf]

Figure S2E

45µg total lysate / sample

Membrane 1

Lane 1 : Ladder
Lane 2: WT
Lane 3: hTERT
Lane 4: hTERT + LT
Lane 5: hTERT + LT + Ras

OXPHOS cocktail

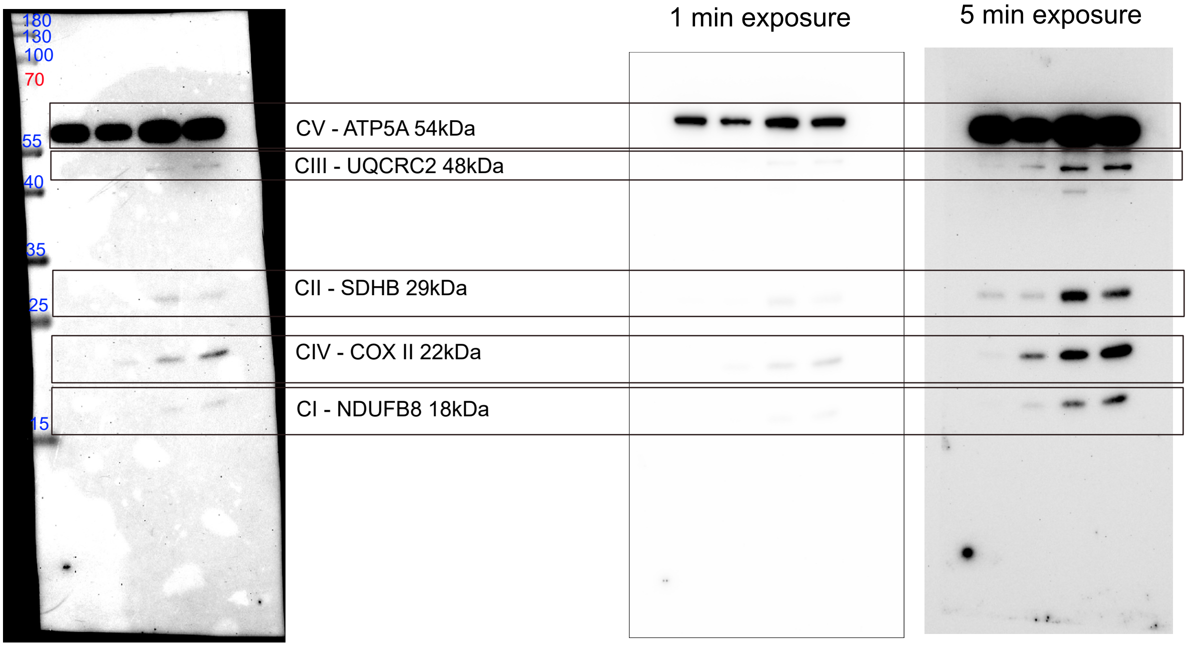

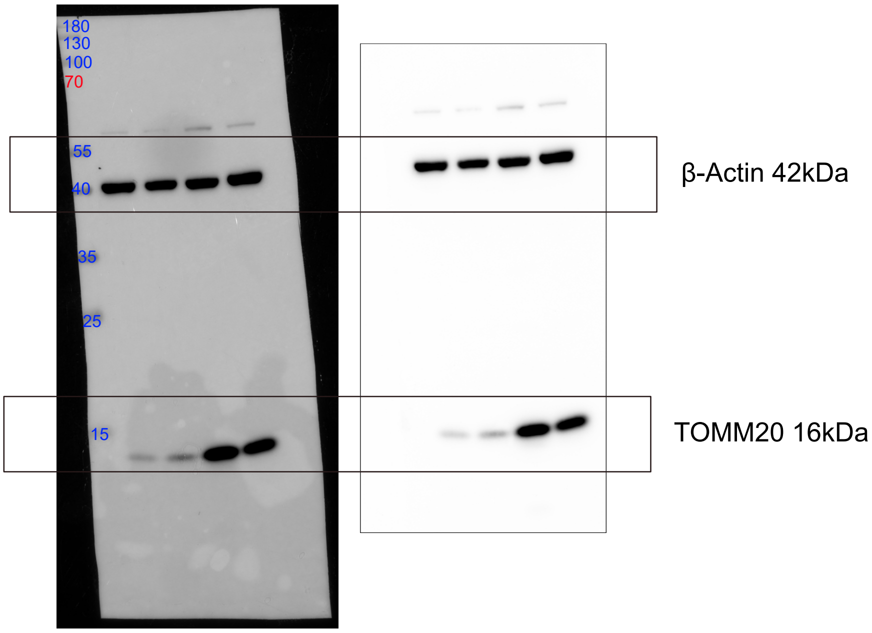

Figure S2E

45µg total lysate / sample

Membrane 2

Lane 1 : Ladder
Lane 2: WT
Lane 3: hTERT
Lane 4: hTERT + LT
Lane 5: hTERT + LT + Ras

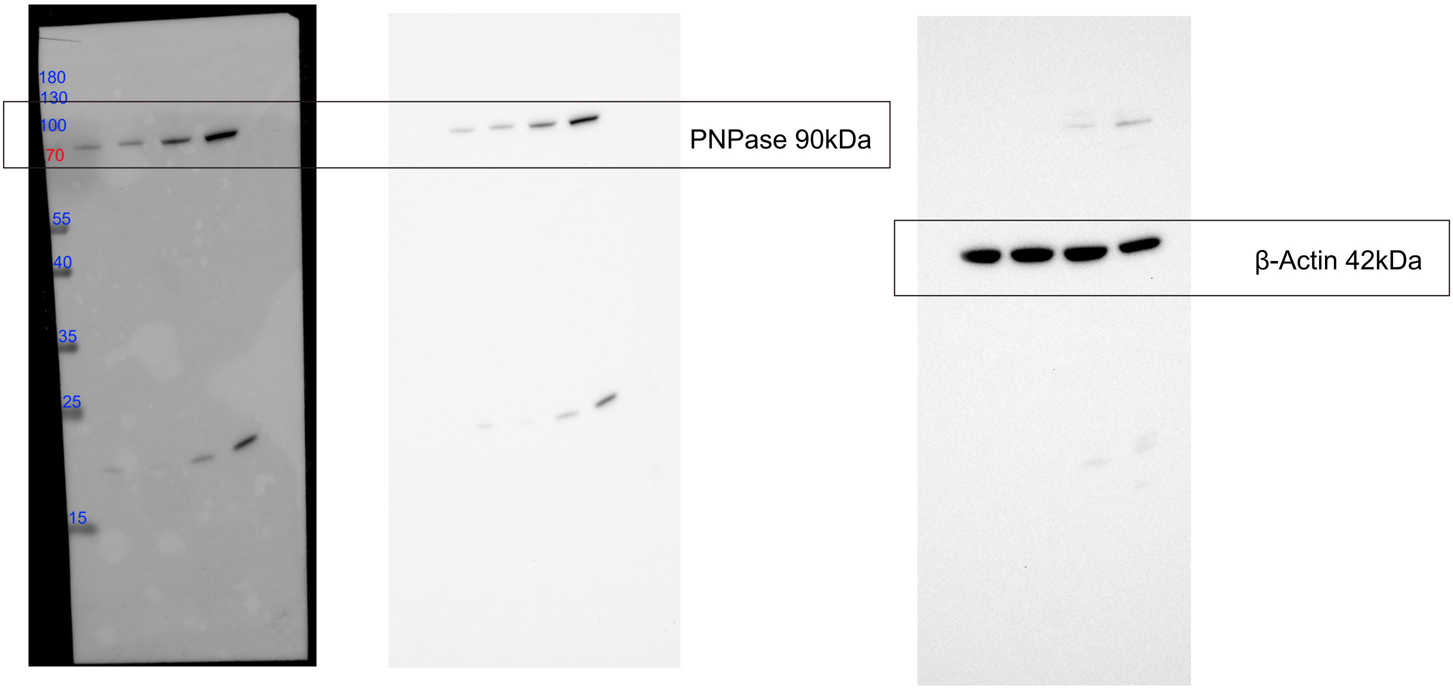

Figure S2E

45μg total lysate / sample

Membrane 3

Lane 1 : Ladder
Lane 2: WT
Lane 3: hTERT
Lane 4: hTERT + LT
Lane 5: hTERT + LT + Ras

GRSF1 53kDa

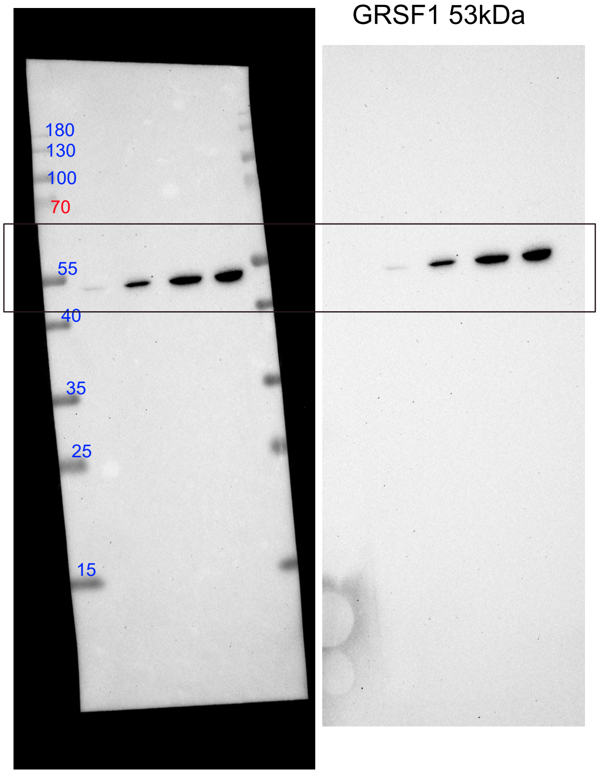

β-Actin 42kDa

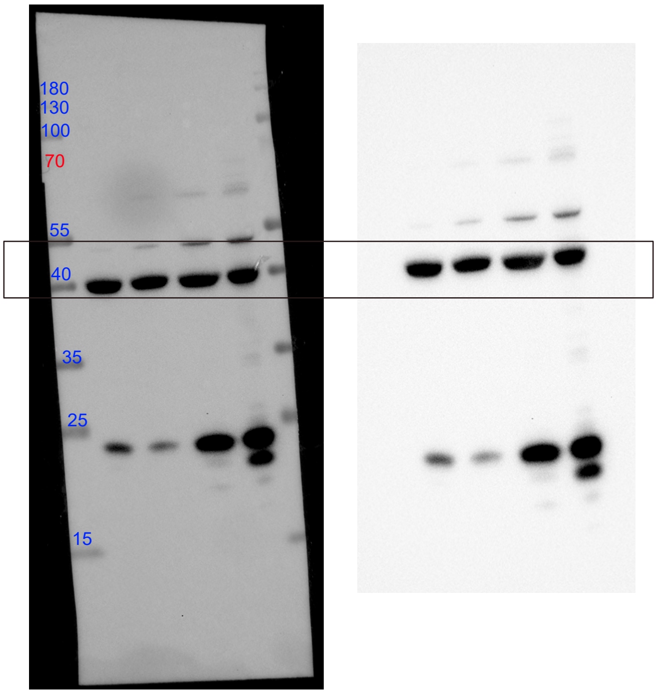

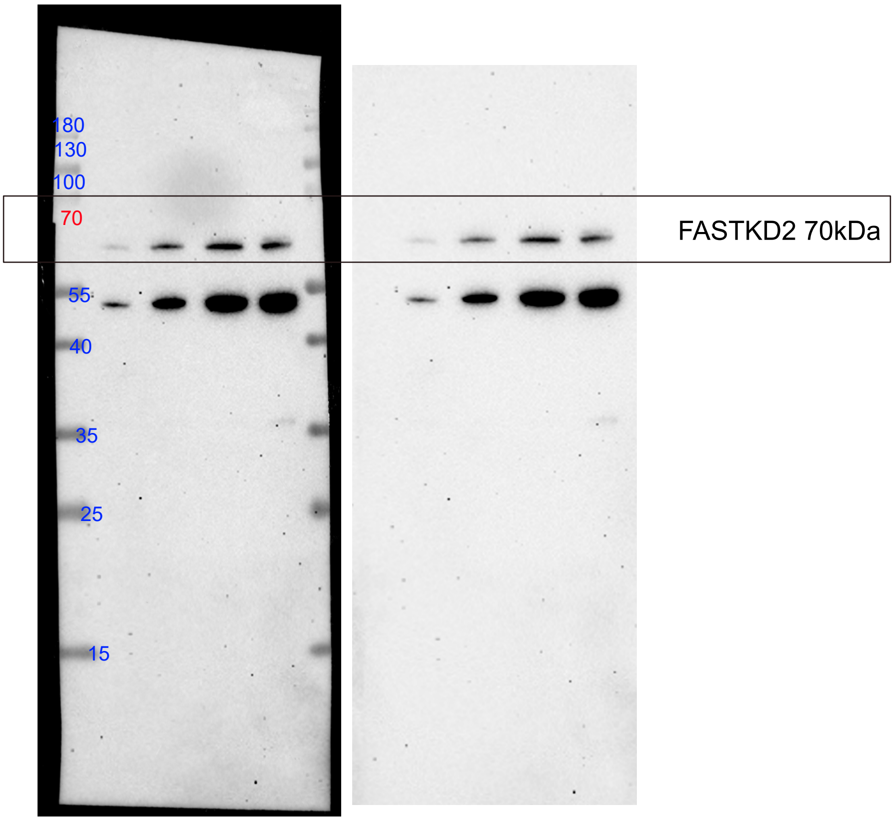

Figure S2E

45µg total lysate / sample

Membrane 4

Lane 1 : Ladder
Lane 2: WT
Lane 3: hTERT
Lane 4: hTERT + LT
Lane 5: hTERT + LT + Ras

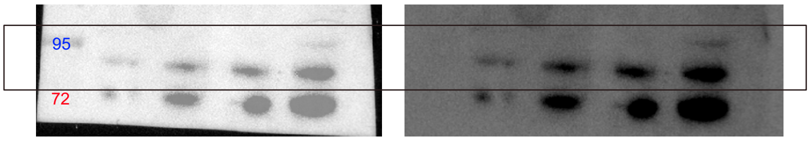

SUV3 90kDa

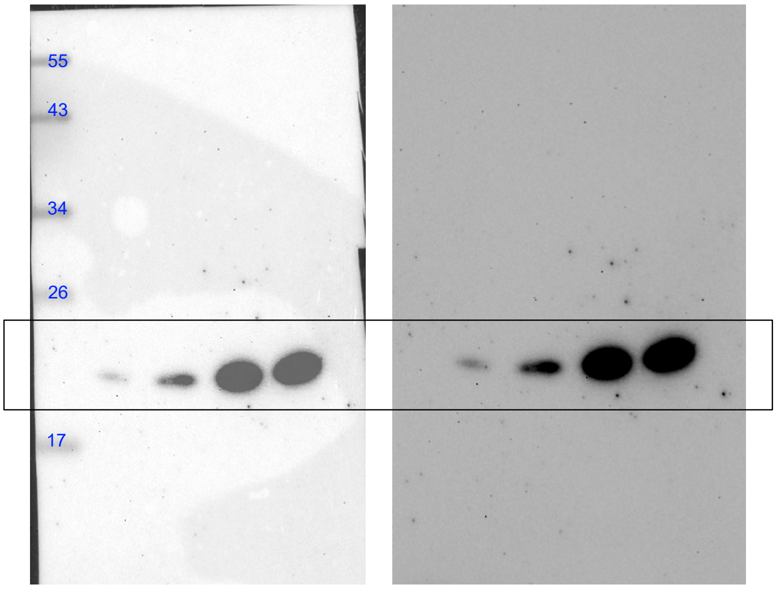

NME6 21kDa

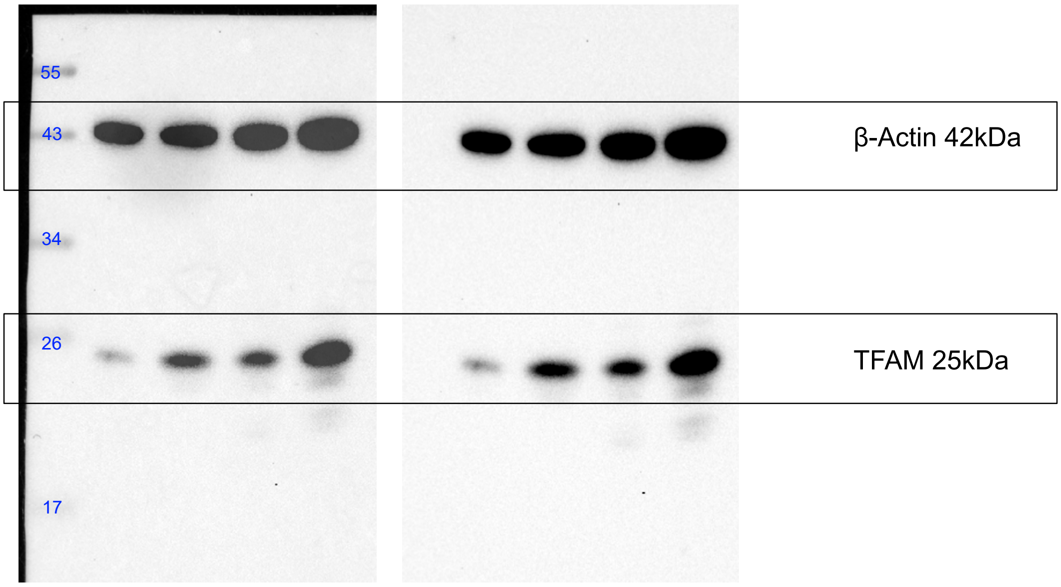

β-Actin 42kDa

TFAM 25kDa
