# Supplementary material for: Mitochondrial double-stranded RNA homeostasis depends on cell-cycle progression
Source: Life Sci Alliance. 2024 Aug 29;7(11):e202402764. doi: 10.26508/lsa.202402764 (PMC11361371; doi:10.26508/lsa.202402764)

Figure 2

Northern Blot

Membrane 1

10µg total RNA / sample

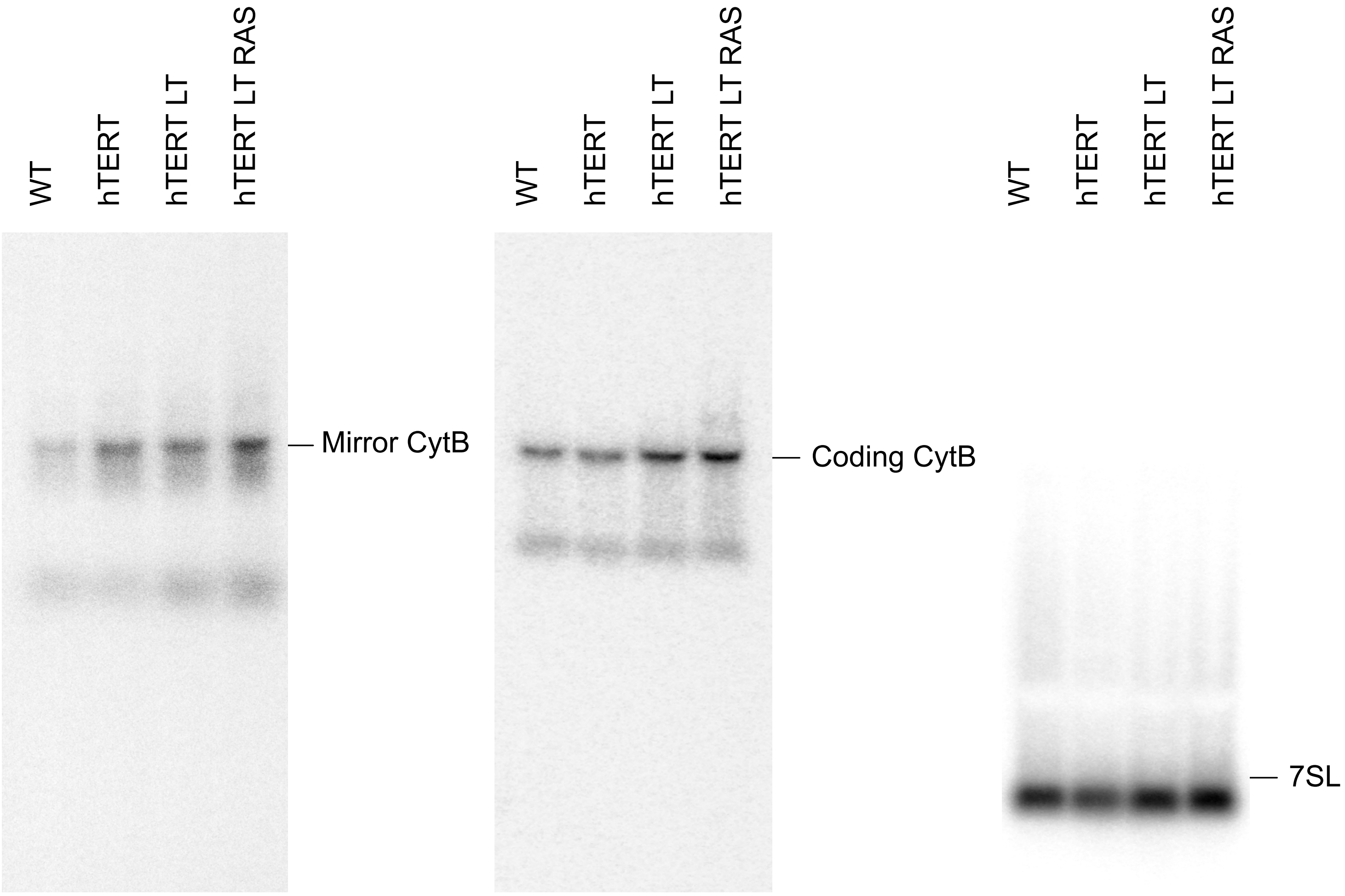

Figure 2

Northern Blot

Membrane 2

10μg total RNA / sample

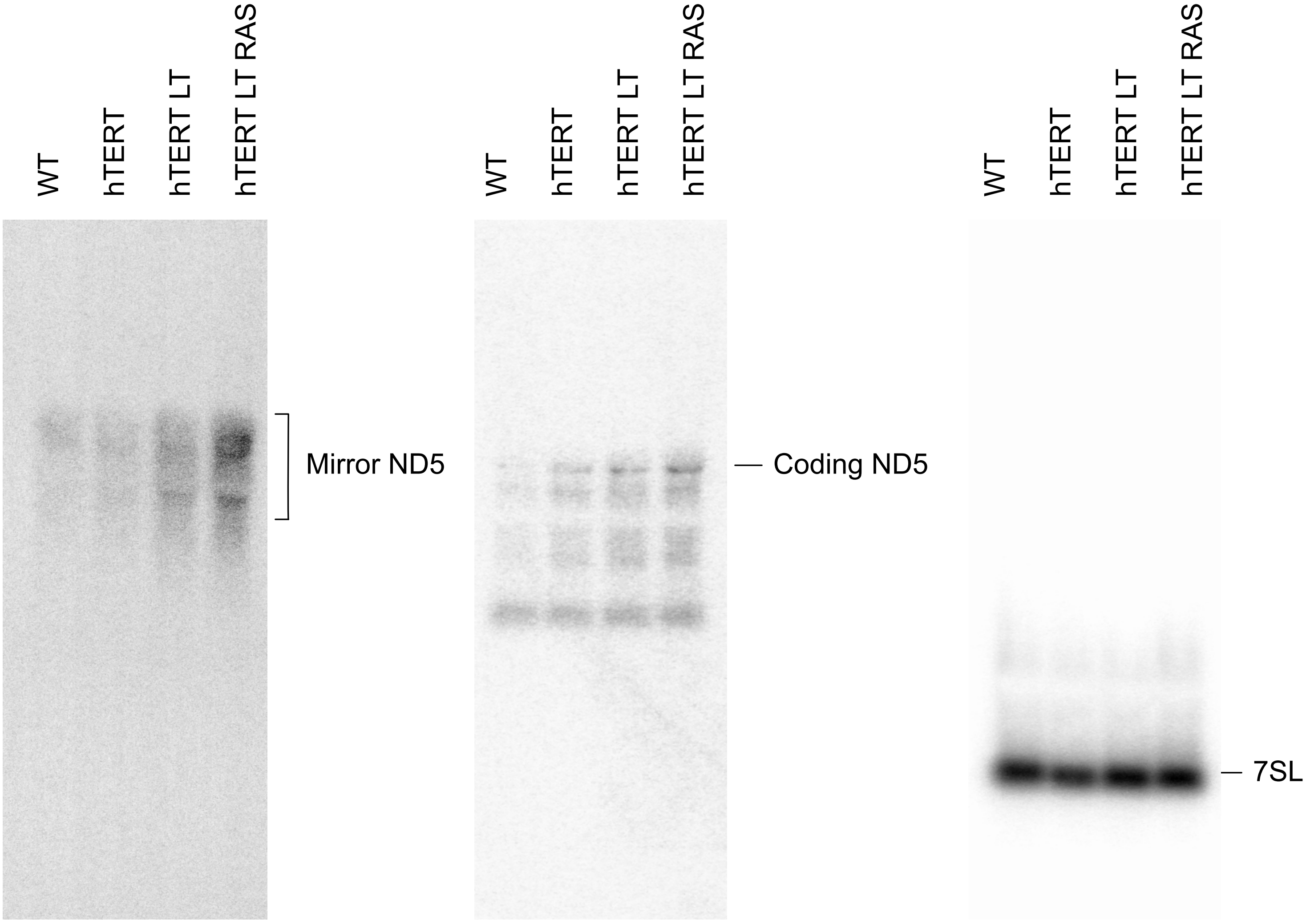

Supplement: Supplementary file 3 [file LSA-2024-02764_SdataF2.pdf]
