# Supplementary material for: Mitochondrial double-stranded RNA homeostasis depends on cell-cycle progression
Source: Life Sci Alliance. 2024 Aug 29;7(11):e202402764. doi: 10.26508/lsa.202402764 (PMC11361371; doi:10.26508/lsa.202402764)

Figure 4G
50µg total protein / sample

Membrane 1

SUV3 (1:1000) Atto 30sec

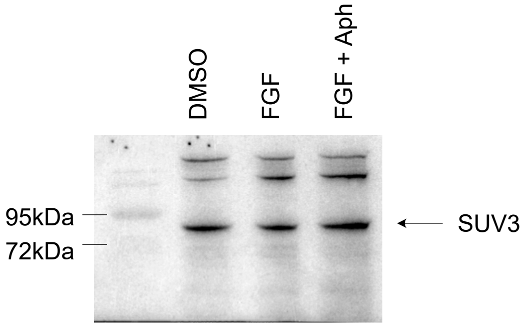

PNPase (1:1000) Plus 30sec

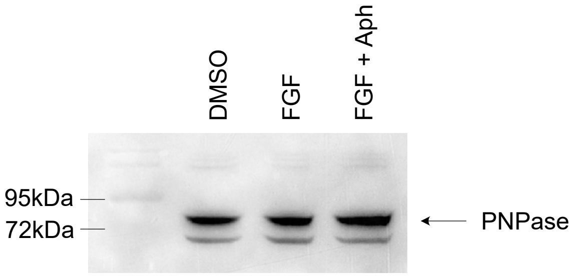

POLRMT (1:1000) Femto 2mins

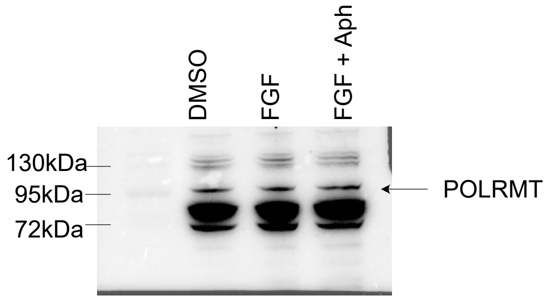

B-Actin (1:30000) Plus 30sec

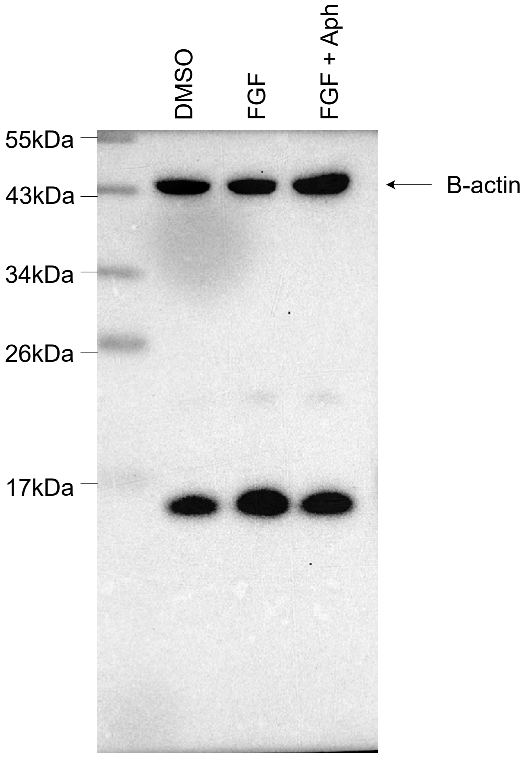

Figure 4G
50µg total protein / sample

Membrane 2

FASTKD2 (1:1000) Plus 1min

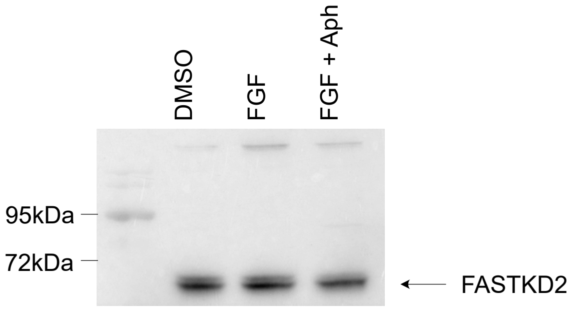

B-Actin (1:30000) Plus 30sec

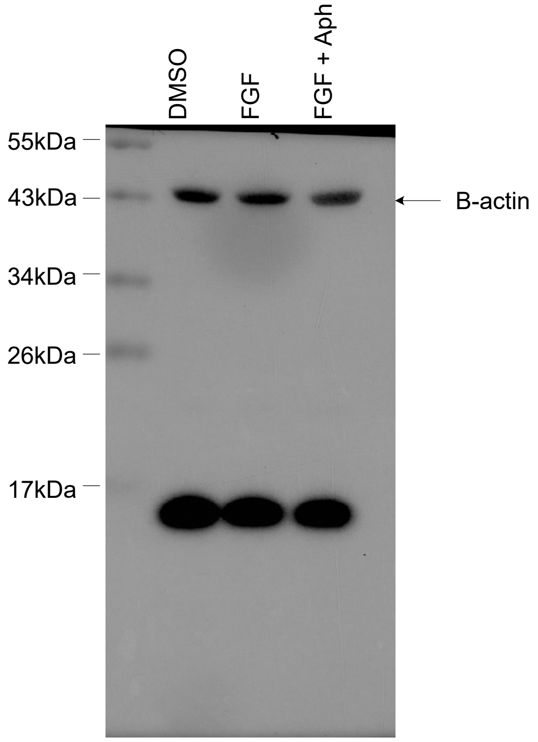

TOMM20 (1:1000) Plus 1min

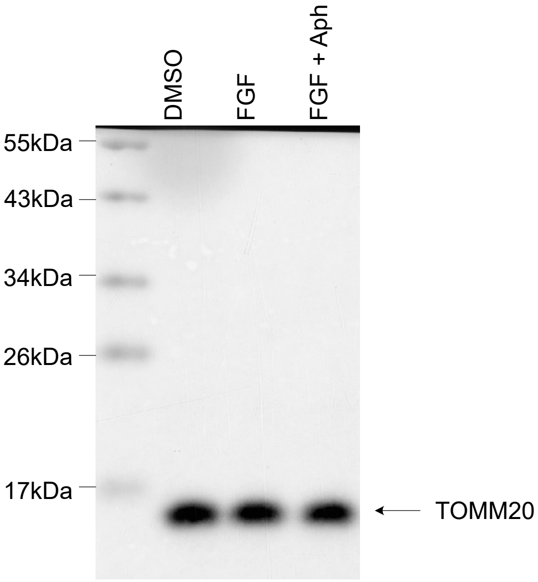

NME6 (1:1000) Femto 3min

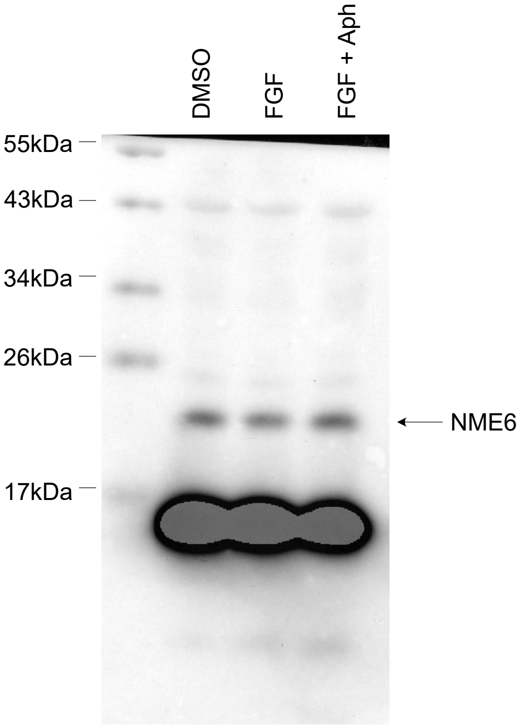

Supplement: Supplementary file 4 [file LSA-2024-02764_SdataF4.pdf]
